# Targeting Stereotactic Body Radiotherapy on Metabolic PET- and Immuno-PET-Positive Vertebral Metastases

**DOI:** 10.3390/biomedicines8120548

**Published:** 2020-11-28

**Authors:** Baptiste Pichon, Caroline Rousseau, Audrey Blanc-Lapierre, Gregory Delpon, Ludovic Ferrer, Vincent Libois, Matthieu Le Turnier, Cédric Lenoble, Caroline Bodet-Milin, David M. Goldenberg, Françoise Kraeber-Bodere, Stéphane Supiot

**Affiliations:** 1 Department of Radiotherapy and Nuclear Medicine Institut de Cancérologie de l’Ouest (ICO), F-44800 Saint-Herblain, France; bpichon@clinique-pasteur.com (B.P.); Caroline.Rousseau@ico.unicancer.fr (C.R.); Audrey.Blanc-Lapierre@ico.unicancer.fr (A.B.-L.); Gregory.Delpon@ico.unicancer.fr (G.D.); ludovic.Ferrer@ico.unicancer.fr (L.F.); vincent.libois@ico.unicancer.fr (V.L.); francoise.bodere@chu-nantes.fr (F.K.-B.); 2 Department of Radiation Oncology, Clinique Pasteur, 31300 Toulouse, France; 3 Centre de Recherche en Cancéro-Immunologie Nantes Angers (CRCINA), Inserm U1232, CNRS ERL 6001, Université de Nantes, F-44000 Nantes, France; caroline.milin@chu-nantes.fr; 4 Department of nuclear medicine and radiology, Centre Hospitalier Universitaire, F-44000 Nantes, France; matthieuleturnier@hotmail.fr (M.L.T.); cedric.lenoble@chu-nantes.fr (C.L.); 5 Center for Molecular Medicine and Immunology, Mendham, NJ 07945-2704, USA; davidgoldenberg38@gmail.com

**Keywords:** immuno-PET, CEA, bispecific antibody, stereotactic body radiation therapy (SBRT), vertebral metastases, PET-CT, targeting, CTV, GTV, radiation oncology, stereotactic ablative body radiotherapy (SABR)

## Abstract

(1) Background: Stereotactic body radiotherapy (SBRT) for vertebral metastases (VM) allows the delivery of high radiation doses to tumors while sparing the spinal cord. We report a new approach to clinical target volume (CTV) delineation based on anti-carcinoembryonic antigen (CEA) positron emission tomography (pretargeted immuno-PET; “iPET”) in patients with metastatic breast cancer (BC) or medullary thyroid cancer (MTC). (2) Methods: All patients underwent iPET, spine magnetic resonance imaging (MRI), and positron emission tomography-computed tomography (PET-CT) using ^18^F-deoxyglucose (FDG) for BC or ^18^F-dihydroxy-phenylalanine (F-DOPA) for MTC. Vertebrae locations and vertebral segments of lesions were recorded and the impact on CTV delineation was evaluated. (3) Results: Forty-six VM eligible for SBRT following iPET were evaluated in eight patients (five BC, three MTC). Eighty-one vertebral segments were detected using MRI, 26 with FDG or F-DOPA PET/CT, and 70 using iPET. iPET was able to detect more lesions than MRI for vertebral bodies (44 vs. 34). iPET-based delineation modified MRI-based CTV in 70% (32/46) of cases. (4) Conclusion: iPET allows a precise mapping of affected VM segments, and adds complementary information to MRI in the definition of candidate volumes for VM SBRT. iPET may facilitate determining target volumes for treatment with stereotactic body radiotherapy in metastatic vertebral disease.

## 1. Introduction

Stereotactic body radiation therapy (SBRT) has emerged as a treatment option for selected patients with VM, with the objectives of pain-relieving decompression, tumor stabilization, and prevention of fractures [1,2]. SBRT for vertebral metastases (VM) is well-tolerated [3] and achieves high rates of local tumor control [4]. The volume of the lesion to be irradiated is defined according to international consensus recommendations for radiosurgery [5] on the basis of MRI, the best anatomical imaging method for the detection of VM [6]. The consensus recommendations suggest that the clinical target volume (CTV) should include abnormal marrow signals suspicious for microscopic invasion and an adjacent normal bony expansion to account for subclinical tumor spread.

Functional imaging using positron emission tomography combined with computed tomography (PET/CT) using, for example, ^18^fluorodeoxyglucose (FDG) has been developed with the objective of improving diagnostic precision for the detection of bone metastases, with a good sensitivity and specificity except for lesions measuring less than 10 mm [7]. In recent years, new markers targeting particular antigens of tumor cells were designed to overcome the limitations of FDG. A promising option to improve diagnostic imaging has been immuno-PET, which combines the high sensitivity of PET with the specificity and selectivity of a monoclonal antibody against a given tumor cell-surface marker. Immuno-PET (iPET) imaging has been shown to be clinically effective in the diagnosis and staging of cancer [8]. Carcinoembryonic antigen (CEA) represents an attractive target in several cancers as it is highly expressed in breast cancer (BC) [9], medullary thyroid cancer (MTC) [10] and colorectal cancer [11]. Another use of iPET may be to exploit the specificity of its imaging to define the target tumor volume for SBRT. With the development of the linear accelerator combined with PET/CT, it is of major importance to study the impact of PET/CT imaging on the definition of SBRT volumes [12,13].

The objective of this study was to evaluate a new method for the delineation of VM CTVs based on pretargeted anti-CEA iPET, compared with reference MRI images and conventional functional PET/CT imaging, in patients with medullary thyroid carcinoma (MTC) or breast cancer (BC).

## 2. Materials and Methods

### 2.1. Patient Population

This study is an ancillary investigation performed in patients with metastatic BC or MTC and VM included in two trials (NCT01730612 and NCT01730638) [9,10] All patients signed written informed consent declarations. The trial sponsored by Nantes University Hospital was approved on 7/11/2012 by the responsible ethics committee (Comité de Protection des Personnes Ouest IV-Nantes). Patients with metastatic MTC or HER2-negative BC were included. The vertebrae for which SBRT was not indicated (circumferential lesions, signs of cord compression or epidural extension) were excluded.

#### 2.1.1. Breast Cancer Cohort

Patients ≥ 18 years, with progressive HER2-negative metastatic BC, after standard treatments and presenting with a CEA serum level ≥ 10 ng/mL, and with at least one lesion ≥ 10 mm on conventional imaging (CT, MRI, and bone scan), were eligible. The other inclusion criteria were Karnofsky performance status ≥ 70, minimum life expectancy of six months, creatinine ≤ 2.5 × normal, normal serum human antimouse antibody (HAMA) and human antihuman antibody (HAHA) titers. The exclusion criteria were pregnancy and breast feeding, any serious active disease or comorbid medical condition, any history of another cancer during the last five years, with the exception of nonmelanoma skin tumors or stage 0 (in situ) cervical carcinoma, and a known hypersensitivity to antibodies or proteins. In the four weeks preceding immuno-PET, a staging workup that included a complete history, physical examination, CEA and CA15-3 serum level measurements were performed.

#### 2.1.2. Medullary Thyroid Cancer Cohort

We recruited patients (age ≥ 18 years) with a diagnosis of MTC presenting a calcitonin serum level of 150 pg/mL or more with at least one metastatic lesion on conventional imaging (10 mm or greater). The other inclusion criteria were an Eastern Cooperative Oncology Group performance status of 0–1 or Karnofsky performance status of 70 or greater, creatinine 2.5 or less, minimum life expectancy of six months, and normal serum HAMA and HAHA titers. The exclusion criteria were any history of other cancer during the last five years with the exception of nonmelanoma skin tumors or stage 0 (in situ) cervical carcinoma, breast feeding and pregnancy; anticancer treatment within six weeks before iPET or necessity to start anticancer treatment in the three months after iPET; any serious active disease or comorbid medical condition; and a known hypersensitivity to antibodies or proteins.

### 2.2. PET/CT and MRI Imaging

PET images were interpreted by consensus of two independent nuclear medicine physicians (CR, FKB and CBM). MRI images were analyzed by two independent radiologists (VL, ML, CL).

#### 2.2.1. PET/CT Imaging

For FDG in BC patients, a whole body PET-CT scan was performed one hour after injection (3–4 MBq/kg IV). For ^18^F-dihydroxy-phenylalanine (F-DOPA) in MTC patients, the acquisition started five minutes after injection of tracer (3–4 MBq/kg IV) centering on the metastatic area for ten minutes and one hour after injection, a whole body PET-CT was performed.

PET/CT was performed using a four-ring Siemens Biograph mCT system with time-of-flight capability 60 and 120 min after ^68^Ga-IMP288 injection and reconstructed using a three-dimensional ordinary Poisson ordered-subset expectation maximization with point-spread function correction and time-of-flight mode (three iterations, 21 subsets, 2 mm in full width at half maximum Gaussian post-filtering, and voxel size of 4 × 4 × 2 mm). Whole-body images were acquired under normal tidal respiration for 2.5 min per bed position. CT was performed using variable mAs, 120 kVp, and a pitch of 1 without contrast enhancement. Images were acquired from the top of the head to mid-thigh (six–eight steps per patient). Tumor SUVmax (T-SUVmax) was determined on the most intense focus in the whole-body scan. Tumor burden was analyzed using total tumor volume (TTV).

#### 2.2.2. iPET-CT Imaging

The reagents were prepared for human use by Immunomedics, Inc. (Morris Plains, NJ, USA). A 1.85-GBq (at calibration time) pharmaceutical-grade gallium-68 generator (Eckert-Ziegler, Germany) was used. ^68^Ga-IMP288 was obtained with a specific activity of 40 to 100 MBq/nmol, with a radiochemical purity > 95%.

TF2, an engineered trivalent BsMAb composed of a humanized anti–histamine-succinyl-glycine Fab fragment derived from the murine 679 antibody and 2 humanized anti-CEA Fab fragments derived from the hMN-14 antibody, was diluted in 250 mL 0.9% NaCl and ^68^Ga-IMP288 in 50 mL of 0.9% NaCl were administered by I.V. infusion over 30 min. Patients were premedicated with oral antihistamine the day before TF2 infusion and with I.V. 5 mg anti-histamine (polaramine) and 500 mg corticosteroid (hydrocortisone hemisuccinate) 5 min before infusion of 60 to 120 nmol of TF2, and 3 to 6 nmol of ^68^Ga-IMP288 administered 24 to 42 h later.

iPET abnormal uptake was defined visually as focal increase of uptake higher than the surrounding background. For skeletal and liver lesions, if more than 10 lesions per bone and similarly for the entire liver were counted, the lesion number was nevertheless capped at ten.

CT and bone MRI were analyzed by consensus of two radiologists with expertise in oncology blinded to the other diagnostic results, and FDG-PET and immuno-PET were analyzed by consensus of two nuclear medicine physicians with expertise in immunotargeting and PET. For both ethical and practical reasons, not every suspected lesion was evaluated by histology. Complementary imaging, primarily MRI, was performed to assess the most important lesions suspected by immuno-PET and not detected by the initial work-up (CT and bone MRI). The gold standard was therefore determined on the basis of histology and imaging follow-up. Indeed, FDG-PET, CT, pelvic- spinal MRI, and any added imaging were performed three months after iPET to confirm the abnormalities.

#### 2.2.3. MRI Acquisition

MRI images were obtained with sagittal cuts for the entire spinal column with T1-weighting and STIR. For some patients, axial images were also obtained with T1-weighting, injection of gadolinium contrast, FAT SAT and T2. MRI images were obtained on a 1.5T Siemens AERA (Siemens, Knoxville, TN, USA).

### 2.3. Analysis of CTVs Determined by MRI and PET and Determination of Sensitivity

Analyses were made of the volumes for each positive vertebra on iPET, distinguishing the affected vertebrae, and the number and localization of the affected vertebral segments. For conventional PET/CT and iPET imaging, segmentation was automatically defined by isocontour at 40% of the SUVmax using Oncoplanet software (DOSIsoft 3.1, Cachan, France). MRI tumor volumes were defined as T1 hyposignal and a T2 and STIR hypersignal lesions. For each image, a clinical target volume (CTV) was defined using the International Spine Radiosurgery Consortium (ISRC) six segments schema [5] and compared with the results from the other imaging modalities (conventional PET/CT, MRI and iPET). True-positive (TP) results corresponded to an abnormal image by a scanning method. A negative finding on an imaging study was considered false-negative (FN) if positive by one other imaging modality. Percent sensitivity [(TP/TP + FN) × 100] on a vertebral segment basis was calculated for each imaging procedure.

### 2.4. Statistical Methods

The continuous variables (Tumor SUVmax and TTV) were described by median and interquartile range (IQR). Between groups differences in numbers of detected lesions were assessed using the Chi square test or the Fisher’s exact test. Proportions were reported with their 95% confidence intervals (CI) calculated using the binomial distribution approach. Variable comparisons between FDG-PET and immuno-PET were performed by means of the Wilcoxon signed-rank test. All comparisons were two-sided, with a significance limit < 5%. All calculations were made using the Stata SE 13.1 statistical tool (StataCorp LP, College Station, TX, USA).

## 3. Results

### 3.1. Patient Population and Number of Vertebrae

**Figure 1:**
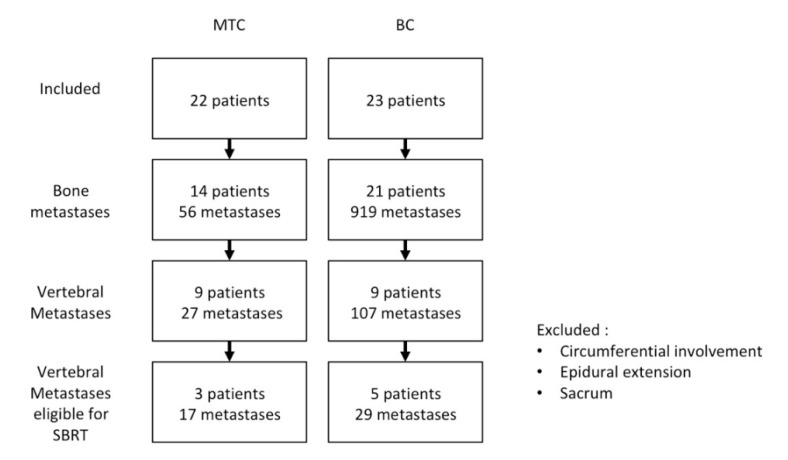
Trial flow chart. Abbreviations: MTC, medullary thyroid carcinoma; BC, breast cancer.

In total, 45 metastatic patients expressing CEA were included (23 BC, 22 MTC, Figure 1) among which 18 patients (nine BC, nine MTC) with VM were selected. In MTC patients, the median calcitonin concentration was 850 pg/mL (range, 154–39,290). In BC and MTC patients, the median CEA concentration was 73.45 ng/mL [range 35.1–111.8] and 22.5 ng/mL (range, 3–1443) respectively. In BC patients, tumor uptake was not significantly different between iPET and FDG-PET, with a median SUVmax of 23.83 [IQR: 9.09–44.65] and 15.87 [interquartile range IQR: 11.70–18.87], respectively (*p* = 0.088). In MTC patients, median SUVmax was 19.54 [range 4.09–94.14] and 2.79 [range 1.72–57.24] for iPET and F-DOPA respectively. Tumor burden evaluated by functional volumes was equivalent between both iPET (median TTV 294 [IQR: 159–558]) and FDG-PET (median TTV 299 [IQR: 139–410] for FDG-PET) (*p* = 0.256).

One hundred-and-thirty-four lesions were detected by iPET. We excluded sacral VM and nonsacral VM ineligible to SBRT due to a circumferential involvement or an epidural extension. Eight patients (5BC, 3 MTC) were selected as having 46 VM eligible to SBRT (29 for BC and 17 for MTC; 3 cervical, 26 thoracic and 17 lumbar; Table 1). Only 35 of these 46 VM were positive in MRI. Respectively 15 and 1 VM were detected on FDG or F-DOPA PET/CT.

### 3.2. Vertebral Segments Analysis

**Figure 2:**
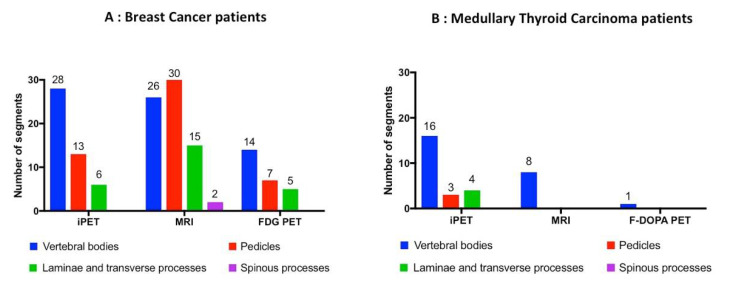
Distribution of detected lesions within vertebrae segments in breast cancer (**A**) and medullary thyroid carcinoma (**B**) patients. Blue: vertebral bodies; red: pedicles; green: laminae and transverse processes; purple: spinous processes.

**Figure 3:**
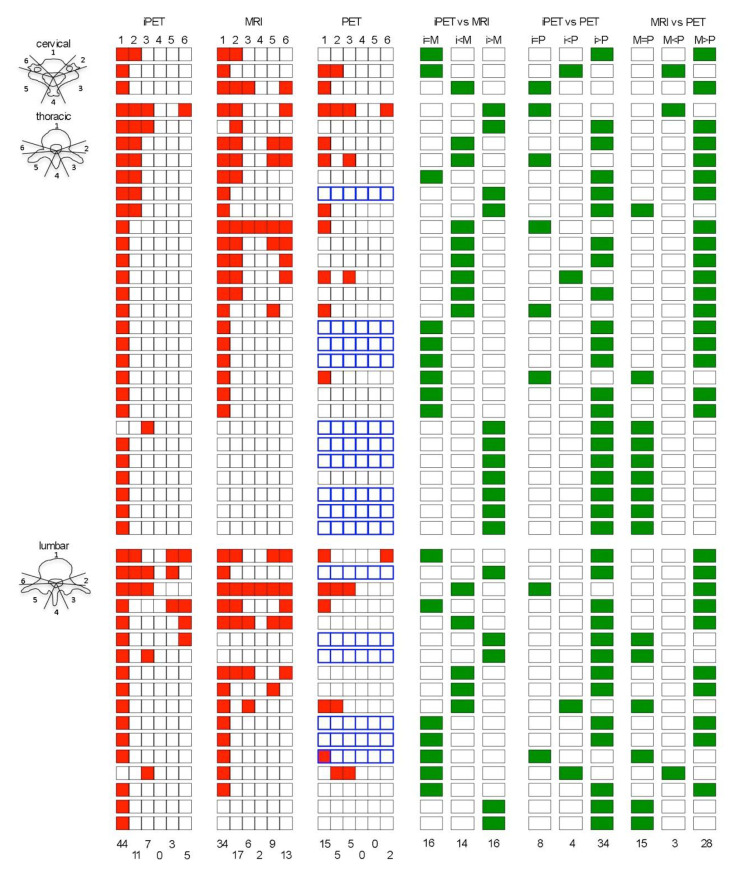
Schematic distribution of each vertebrae segments determined in iPET, MRI, and PET, and comparison of the number of segments determined by each imaging modality at the cervical, thoracic and lumbar level. 1: vertebral body; 2: left pedicle; 3: left transverse process and lamina; 4: spinous process; 5: right transverse process and lamina; 6: right pedicle; red square = involved segment; in the PET column, blue squares stand for F-DOPA PET/CT, the others stand for FDG PET/CT; M = MRI; i = iPET; P = PET/CT; green square = result of the comparison of the number of segments determined by each imaging modality.

**Figure 4:**
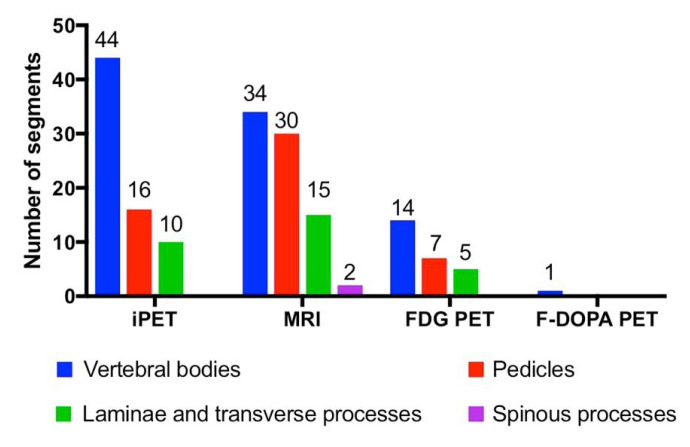
Distribution of detected lesions within vertebral segments in all patients. Blue: vertebral bodies; red: pedicles; green: laminae and transverse processes; purple: spinous processes. iPET: immune Positron Emission Tomography. MRI: Magnnetic Resonance Imaging. PDG-PET: Fluorodeoxyglucose Positron Emission Tomography; F-DOPA PET: ^18^F-dihydroxy-phenylalanine Positron Emission Tomography.

MRI identified more vertebral segments than iPET and FDG- or F-DOPA-PET/CT (81, 70 and 27 respectively, Table 2, Figure 2, Figure 3 and Figure 4). iPET was able to detect more lesions than MRI in vertebral bodies (44/46 vs. 34/46, *p* < 0.01), but fewer in pedicles (16/37 vs. 30/37, *p* < 0.01) and a similar number in laminae or transverse processes (10/22 vs. 15/22, *p* = 0.13), and spinous processes (0/2 vs. 2/2, *p* = 0.33) (Figure 2). FDG-PET detected 26 segments out of 86 in BC patients. F-DOPA detected only one segment in a MTC patient. One hundred and nine segments were considered as positive using all imaging modalities (86 and 23 in BC and MTC patients, respectively). The sensitivity to predict the involvement of a vertebral segment was 64% (95% CI: 54%–73%), 74% (95% CI: 65%–82%), 30% (95% CI: 21%–41%) and 4% (95% CI: 0%–22%) for iPET, MRI, FDG-PET and F-DOPA PET, respectively.

### 3.3. CTV Delineation

**Figure 5:**
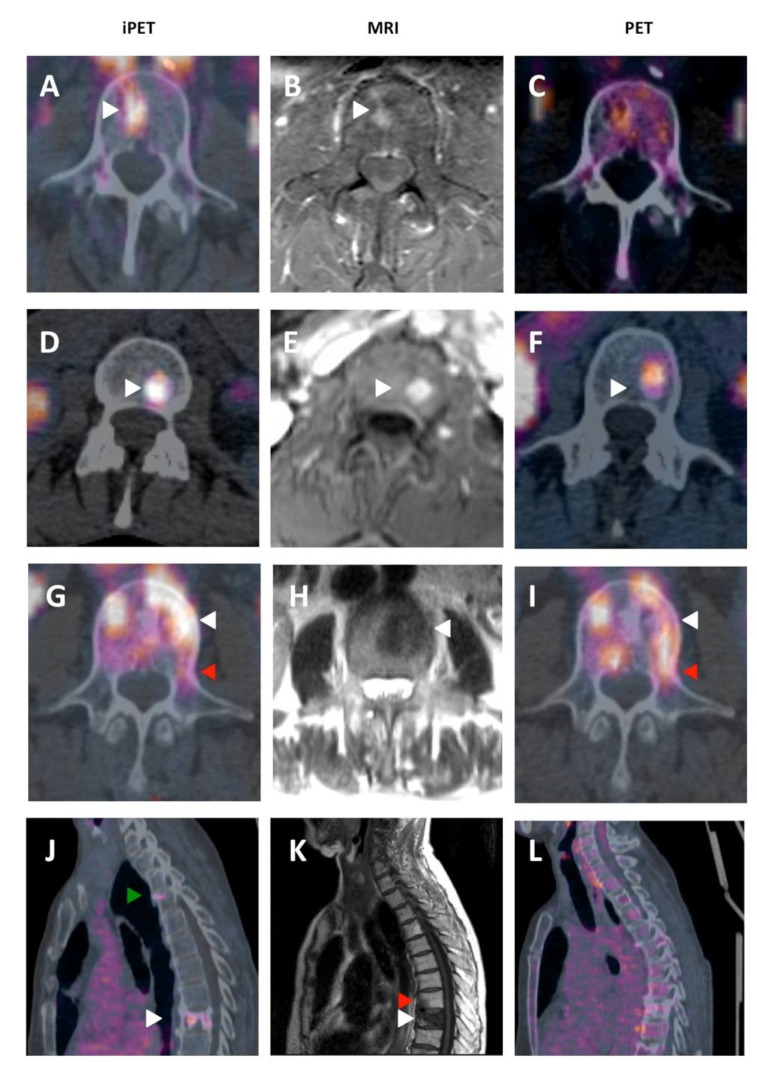
Representative cases of iPET/CT, MRI and PET/CT imaging in VM. (**A**–**C**) 51-year-old female with metastatic breast cancer: L4 spinal metastasis. (**A**) Axial iPET/CT: increased tracer uptake in vertebral body (white arrow); (**B**) axial MRI with T1 weighting and injection of gadolinium contrast: hypersignal lesion in vertebral body (white arrow). (**C**) Axial PET-CT ^18^FDG: background tracer uptake, no visible lesion. (**D**–**F**) 46-year-old female with metastatic medullary thyroid carcinoma: L3 spinal metastasis. (**D**) Axial iPET/CT: increased tracer uptake in vertebral body (white arrow). (**E**) Axial MRI with T1 weighting and injection of gadolinium contrast: hypersignal lesion in vertebral body (white arrow). (**F**) Axial PET-CT ^18^F-DOPA: increased tracer uptake in vertebral body (white arrow). (**G**–**I**) 47-year-old female with metastatic breast cancer: L3 spinal metastasis. (**G**) Axial iPET/CT: increased tracer uptake in vertebral body (white arrow) and extension in left pedicle (red arrow). Tumor volume diffusely involves the vertebral body and the left pedicle in iPET. The corresponding Clinical Target Volume (CTV) would thus include, according to the International Spine Radiosurgery Consortium (ISRC), the entire vertebral body, the left pedicle, and ipsilateral transverse process and lamina. (**H**) Axial MRI with T2 weighting: hypersignal lesion in vertebral body (white arrow) without extension in left pedicle. This MRI would suggest a CTV that includes the entire vertebral body and the ipsilateral pedicle. (**I**) Axial PET-CT ^18^FDG: increased tracer uptake in vertebral body (white arrow) and extension in left pedicle (red arrow). (**J**–**L**) 57-year-old female with metastatic breast cancer: T2, T8, T9 spinal metastasis. (**J**) Sagittal iPET/CT: increased tracer uptake in T2 (green arrow) and T9 (white arrow) vertebral bodies. The lesion in T8 is not visible. (**K**) Axial MRI with T1 weighting: hyposignal lesion in T8 (red arrow) and T9 (white arrow) vertebral bodies. The lesion in T2 is not visible. (**L**) Sagittal PET-CT ^18^FDG: background tracer uptake, no visible lesion.

In 16/46 VM, a similar number of vertebral segments was determined in iPET and MRI, but in only 14/46 (30%) the CTV was strictly identical between the two modalities, which means that iPET-based delineation modified MRI-based CTV in 70% (32/46) of cases (Figure 3). In the 35 VM with at least one positive segment on MRI, the delineation based on iPET enlarged the CTV in 9/35 (26%). In four VM, a similar number of non-null vertebral segments was determined in PET and MRI, but in only three the CTV was identical between the two modalities. In the 35 VM with at least one positive segment on MRI, the delineation based on PET enlarged the CTV in 3/35 (9%). Figure 5 presents examples of MRI, PET, and iPET images in different clinical situations.

## 4. Discussion

We have evaluated the feasibility of targeting metastatic vertebral lesions using metabolic PET and iPET with a view to treatment with SBRT in patients with metastatic CEA-expressing BC and MTC. We have shown that MRI and iPET provided complementary information, which enables better definition of the CTV and, thanks to iPET, the prospect of a new method for the delineation of VM and SBRT.

In the first instance, we have looked for a better characterization of the CTV with the assistance of functional imaging with FDG-PET/CT and F-DOPA-PET/CT. The literature suggests that the sensitivity of FDG-PET in detecting spinal metastases is high [14], but only one study evaluated the usefulness of FDG-PET/CT for radiosurgery planning in patients with recurrent spinal metastasis [15]. In our study, numerous vertebrae, judged pathological by MRI, were not detected by these techniques. Our results suggest that trying to determine the affected segment using FDG-PET/CT and F-DOPA-PET/CT is disappointing and poorly informative, since numerous vertebrae, judged pathological by MRI, were not detected by these techniques.

In the face of this difficulty, iPET, due to its excellent contrast, seems to be an interesting technique to assist with the delineation of target volumes for radiotherapy to secondary bony metastatic lesions in CEA- (or CEACAM5-) expressing tumors. The results seem promising with, in the present case, eleven vertebrae that were positive on iPET that were negative on MRI. This shows pathological vertebrae expressing CEA may not be visible on MRI, especially in oligometastatic disease.

Having evaluated the feasibility of iPET in the detection of VM, we have concentrated on each pathological vertebra in order to determine precisely the affected zone and its corresponding CTV with a view to treatment with SBRT. We showed that iPET modified the MRI-defined spinal CTV in seven cases out of 10, by enlarging the CTV in more than one in four cases. However, iPET sensitivity and specificity have yet to be fully determined. There may be secondary lesions shown on MRI which, because they do not express CEA, would be invisible on iPET [9]. Even if imaging specificity requiring histological confirmation of false-positive lesions was not determined in this cohort of patients with diffuse disease, iPET is expected to have a high specificity. CEA (specifically the CEACAM5 targeted here) is expressed in 60–80% of metastatic breast cancers [16], and MTC is characterized by intense expression of CEA [17]. Anti-CEA iTEP could also be applicable to any CEA-expressing cancer, such as colorectal, urinary bladder, or lung cancers.

Our studies have certain limitations, since patient selection was on the basis of iPET and not MRI, which is the gold standard for the detection of VM. This choice was made on the basis that the investigation is rarely available and the protocol-based nature of this kind of imaging. Although this was a small study in terms of patient numbers, the number of lesions positive on iPET was significant, and enabled us to have samples of VM sufficient to make comparisons between our results with MRI and PET.

## 5. Conclusions

iPET, a new technique for phenotype tumor imaging, allows a precise mapping of affected VM segments, and adds complementary information to MRI in the definition of candidate volumes for VM SBRT. iPET may facilitate determining target volumes for treatment with SBRT in metastatic vertebral disease. These results warrant further clinical evaluation.

## Figures and Tables

**Table 1:** Localization of pathological vertebrae eligible for SBRT. BC: breast cancer; MTC: medullary thyroid carcinoma; NA: not applicable. iPET: immune Positron Emission Tomography. MRI: Magnnetic Resonance Imaging. PDG-PET: Fluorodeoxyglucose Positron Emission Tomography; F-DOPA PET: ^18^F-dihydroxy-phenylalanine Positron Emission Tomography.

		iPET	MRI	FDG-PET	F-DOPA PET
BC	Cervical	3	3	2	NA
	Thoracic	16	15	8	NA
	Lumbar	10	9	5	NA
MTC	Cervical	0	0	NA	0
	Thoracic	10	4	NA	0
	Lumbar	7	4	NA	1
Total		46	35	15	1

**Table 2:** Number of vertebral segments determined in MRI, PET and iPET and sensitivity of each imaging modality BC: breast cancer; MTC: medullary thyroid carcinoma; NA: not applicable. iPET: immune Positron Emission Tomography. MRI: Magnnetic Resonance Imaging. PDG-PET: Fluorodeoxyglucose Positron Emission Tomography; F-DOPA PET: ^18^F-dihydroxy-phenylalanine Positron Emission Tomography.

Location		iPET	MRI	FDG-PET	F-DOPA PET	Number of Individual Vertebrae Segments
BC	Total	47	73	26	NA	86
	Cervical	4	7	3	NA	8
	Thoracic	25	38	13	NA	45
	Lumbar	18	28	10	NA	33
MTC	Total	23	8	NA	1	23
	cervical	0	0	NA	0	0
	Thoracic	11	4	NA	0	11
	Lumbar	12	4	NA	1	12
BC + MTC	Total	70	81			109
	Cervical	4	7			8
	Thoracic	36	42			56
	Lumbar	30	32			45
Sensitivity		64% (70/109)	74% (81/109)	30% (26/86)	4% (1/23)	

## Abbreviations

SABR	stereotactic ablative radiotherapy
SBRT	stereotactic body radiation therapy
VM	vertebral metastasis
CTV	clinical target volume
CEA	carcino embryonic antigen
iPET	immuno positron emission tomography
PET-CT	positron emission tomography-computed tomography
BC	breast cancer
MTC	medullary thyroid carcinoma
MRI	magnetic resonance imaging
F-DOPA	l-3,4-dihydroxy-6-[^18^f]fluorophenylalanine
FDG	fluoro desoxy glucose
HAMA	human antimouse antibody
HAHA	human antihuman antibody
IQR	interquartile range
ISRC	International Spine Radiosurgery Consortium
